# Maternal perception of decreased fetal movements from maternal and fetal perspectives, a cohort study

**DOI:** 10.1186/1471-2393-14-286

**Published:** 2014-08-23

**Authors:** Mahdi Sheikh, Sedigheh Hantoushzadeh, Mamak Shariat

**Affiliations:** Maternal, Fetal and Neonatal Research Center, Tehran University of Medical Sciences, Tehran, Iran; Breastfeeding Research Center, Tehran University of Medical Sciences, Tehran, Iran; Maternal, Fetal and Neonatal Research Center, Vali-asr teaching Hospital, Imam Khomeini Hospital Complexes, Keshavarz Blvd, Tehran, 1419733141 Iran

**Keywords:** Feeling, Fetus, Movement, Pregnancy, Sensation

## Abstract

**Background:**

Maternal counting of fetal movement is a popular and valuable screening tool of fetal wellbeing, however it is still not known what percentage of healthy pregnant women who gave birth to healthy term newborns had experienced decreased fetal movements during gestation and what maternal and fetal factors are associated with this maternal perception of decreased fetal movements. The aim of this study was to assess the associations between maternal perception of decreased fetal movements and maternal and fetal factors in normotensive singleton pregnancies with good pregnancy outcome.

**Methods:**

This study was conducted on 729 normotensive singleton pregnant women who had referred for prenatal visit and on follow up gave birth to healthy term newborns. A questionnaire was completed for the participants and ultrasound imaging was performed. Participants were asked to count their fetal movements for one hour/3times/day. Participants were followed till delivery to exclude mothers with preterm and/or small for gestational age delivery from the study.

**Results:**

Perception of decreased fetal movement was independently associated with maternal employment (Odds Ratio (OR), 2.66; 95% Confidence Interval (95% CI), 1.35–5.23), not having daily exercise (OR, 4.38; 95% CI, 1.56-8.08) and maternal supine position (OR, 3.85; 95% CI, 1.71–8.83).

**Conclusions:**

8.1% of healthy pregnant women who have good pregnancy outcome report perception of decreased fetal movement when asked to count their fetal movement in third gestational trimester which is independently associated with maternal employment, supine position on counting and not having daily exercise.

## Background

Maternal counting of fetal movement is an easy, inexpensive and valuable screening tool for fetal well-being that increases maternal-fetal bonding. Sensation of decreased fetal movement (DFM) is a common problem among pregnant women; in Norway, as many as 51% of women report that they were concerned about DFM once or more in pregnancy. Only 4 - 15% of pregnant women contact care providers with such concerns [1].

Some studies indicated that women presenting with DFM are at increased risk of stillbirth, fetal growth restriction, fetal distress and preterm birth [1, 2]. Assessment of fetal wellbeing by counting fetal movements in many studies was associated with a decrease in perinatal mortality and morbidity because a mother’s reaction to DFM assists in the identification of high risk fetuses when it might be possible to save the baby’s life [3]. Some studies argued that DFM is not a useful screening tool and that it has a high failure rate [4].

Research has shown that fetal movements are affected by many factors including amniotic fluid volume [5], placental location [6], fetal presentation [7], and fetal gender [8]. Maternal factors could influence fetal movements; in different studies maternal smoking, primiparity, obesity and acute exercise were associated with DFM [2, 6, 9].

In an extensive search of maternal perception of DFM in uncomplicated pregnancies with desirable pregnancy outcome, we could not find a sufficient number of studies. The percentage of pregnant women who gave birth to healthy term newborns who had experienced DFM during gestation and the reasons for the sensation is unclear. It is important to understand the maternal and fetal factors associated with the perception of DFM in healthy pregnant women with desirable pregnancy outcomes for better interpretation and management of this common concern among pregnant women and to know what factors can cause false positive results for this popular screening tool of fetal well-being. Very few studies have assessed the influence of simultaneous maternal and fetal factors on mother’s self-reports of fetal movement perception. Research is lacking regarding whether fetal and maternal positions affect the maternal perception of fetal movements [10], and there are only a few studies that have conflicting evidence on whether parity, obesity, placental location and amniotic fluid volume affect this perception [10].

We undertook this study to increase knowledge of factors affecting maternal perception of fetal movements from the maternal and fetal perspectives in normotensive singleton uncomplicated pregnant women who gave birth to healthy term newborns to facilitate more accurate interpretations of this important, easy and inexpensive tool for the screening of fetal well-being.

## Methods

### Study population and study design

This study was conducted among pregnant women who were referred for prenatal visits to Vali-Asr Teaching Hospital of the Tehran University of Medical Sciences (a tertiary referral hospital with an annual birth rate of 2200 births), Tehran, Iran, between February 2012 and March 2013. A total of 929 pregnant women agreed to participate in the study, and they were followed until the end of pregnancy. Two hundred were excluded because of one or more of the following criteria: preterm birth, small for gestational age (SGA) delivery, maternal smoking, opiate use, diabetes, hypertension, fetal anomaly or multiple gestations.

Seven hundred and twenty-nine women aged from 16 to 45 years at gestational ages of 28 to 40 weeks completed the study after providing informed consent. This study was approved by the ethics committee of the Tehran University of Medical Sciences. The current study has adhered to the STROBE guidelines for observational studies.

### Data collection

Upon enrolment, a standardized questionnaire was completed for every participant by interviewing them and using their medical records. The questionnaire contained information regarding the demographics, medical, gynecological, obstetrical and social history and inquiries about the time of their first perception of fetal movement, body mass index (BMI) before pregnancy, gestational weight gain, daily exercise and its duration, employment, and total hours spent working per day.

In addition upon enrolment an ultrasound imaging was performed for all the participants to document the gestational age, fetal gender and presentation, placental site, and amniotic fluid index (AFI), which was assessed using a summation of the maximum vertical pockets of fluid in the four quadrants of the uterus [8].

We used the Sadovsky method because of its acceptability and high compliance rate among pregnant women [11]. To obtain more accurate results, upon enrolment all the participants were instructed by the physician about what constitutes a fetal movement (rolling and stretching, simple flutter or kicks, strong jab, startle, and high frequency rapid movements). In addition the participants were instructed how to count the fetal movements, they were asked to empty their bladders then count their fetal movements for one hour 3 times a day, 30–60 minutes after meals in a quiet room, they were also asked to maintain their position, which they could choose freely when counting the fetal movements. The number of perceived fetal movements, maternal position and time of movement counting were recorded in the questionnaires. Due to the limited compliance of the participants we couldn’t ask them to count their fetal movement to the end of pregnancy, therefore we asked the participants to count the fetal movements every day for a period of one week form the day after enrolment. All the pregnant women were followed until delivery, and the birth weights and gestational ages of the newborns were recorded to exclude pregnant women with preterm birth and/or SGA delivery from the study.

The affecting factors were compared in 2 groups: mothers with perception of normal fetal movements (equal to or more than 4 fetal movements per hour) and mothers with perception of DFM (less than 4 fetal movements per hour) [11].

### Statistical analysis

All the statistical analyses were performed using SPSS statistical software (version 18.0.0: PASW). The Chi-squared analysis, Fisher’s exact test, independent-samples T test, One-Way ANOVA, and multivariate logistic regression were used to analyze the correlations and relationships among the variables. Sample size was calculated for a power of 80% and an alpha error of 0.05. Estimated odds ratios (ORs) with 95% confidence intervals (95% CIs) and p value were used to evaluate the statistical significance of the associations and correlations between variables. Additionally, because of the different multiple analyses done on the studied populations, Bonferroni correction for the number of analyses were performed on the p values.

## Results

### Descriptive statistics

A total of nine hundred twenty-nine pregnant women participated in the study, of which two hundred were excluded. Of these women 8 were smokers or used opiates, 36 had diabetes, 50 had hypertension, 13 had a fetal anomaly, 28 had multiple gestations and 65 gave birth to a preterm and/or SGA newborn.

At enrollment, the mean ± standard deviation (SD) for the maternal age was 28.5 ± 5.1 years; for gestational age, 31.5 ± 5.3 weeks; for maternal BMI before pregnancy, 25.1 ± 4.7; for gestational weight gain, 11.2 ± 5.5 kg; for gestational age at first perception of fetal movement, 18.9 ± 3.3 weeks.

One hundred and five women (14.4%) were employed. Of these women, 59 (56.1%) worked 8 hours or more per day. One hundred and twenty-six women had daily exercise (17.3%). The participants reported their position during fetal movement perception as the following: 266 women in left lateral recumbent position (41.6%), 101 in right lateral recumbent position (15.8%), 60 in supine position (9.3%), 212 in sitting position (33.2%), 90 women did not report their position.

In the ultrasound studies, 684 mothers had a normal AFI (96.2%), and 20 had polyhydramnios (AFI of > 25 cm or at > 97.5th percentile) (2.8%), 7 had oligohydramnios (AFI of < 5 cm or at < 2.5th percentile) (0.9%) and 18 did not have the information provided. The placental sites were recorded as the following: 365 mothers had anterior placenta (51.1%), 280 had posterior placenta (39.2%), 69 had other sites of placenta (9.7%) and 15 did not have the information provided. Among the fetuses: 559 had cephalic presentation (78.5%), 133 had breech presentation (18.7%), 20 had transverse presentation (2.8%) and 17 did not have the information provided.

### The association of maternal factors and perception of DFM

In our study, 59 healthy pregnant women (8.1%) reported DFM, which was significantly associated with maternal age older than 35 years (p = 0.007), delayed perception of first fetal movements (later than 19 gestational weeks) (p = 0.007), maternal employment (p = 0.002), work time duration more than 8 hours per day (p = 0.04), and maternal state of not having daily exercise (p = 0.001). DFM was perceived more often in the supine position (p = 0.001). There was a borderline association with a higher maternal BMI before pregnancy (P = 0.06). When Bonferroni correction was made on P values only maternal employment (Bonferroni corrected P value (b.p) = 0.04)), maternal state of not having daily exercise (bp = 0.02) and maternal supine position (b.p = 0.02) remained significantly associated with maternal perception of DFM. We did not find significant associations between decreased perception of fetal movements and parity, gestational age or time of perception (Table 1).

**Table 1:** **The association between maternal factors and decreased perception of fetal movement**

Factors	Mothers with		OR	95% CI
	Perception of DFM (n = 59) N (%)	Perception of NFM (n = 670) N (%)		
Maternal age > 35 years	15 (25.4)	83 (12.4)*	2.41	1.28 – 4.52
Multiparty	29 (49.2)	353 (52.7)	0.86	0.51 – 1.47
Delayed first perception of FM	41 (69.5)	348 (51.9)*	2.1	1.18 – 3.74
Maternal employment	17 (28.8)	88 (13.1)*^†^	2.67	1.46 – 4.9
● Working more than 8 h/D	13/17 (76.4)	46/88 (52.2)*	6.95	2.73 – 24.02
Daily exercise	2 (3.38)	124 (18.5)*^†^	0.15	0.03 – 0.64
Maternal position				
● Right recumbent	6 (10.1)	95 (14.1)	0.87	0.33 – 2.25
● Supine	13 (22)	47 (7)*^†^	3.81	1.74 – 8.3
● Sitting	16 (27.1)	196 (29.2)	1.12	0.55 – 2.26
● Left recumbent^◄^	18 (30.5)	248 (37)	-	-
Time of perception				
● Morning	13 (22)	132 (19.7)	1.37	0.5 – 3.76
● Night	40 (67.7)	444 (66.2)	1.26	0.51 – 3.06
● Afternoon^◄^	6 (10.1)	84 (12.5)	-	-

*: Uncorrected p < 0.05 for the comparison between two groups with and without the specified characteristic, ^†^: Bonferroni corrected p < 0.05 for the comparison between two groups with and without the specified characteristic, ^◄^: reference category, OR: odds ratio, 95% CI: 95% confidence interval, DFM: Decreased fetal movements, NFM: Normal fetal movements, GA: Gestational age, h/D: Hours/Day.

### The association of fetal factors and perception of DFM

Before Bonferroni correction, perception of DFM was significantly associated with female fetuses (p = 0.04), but when Bonferroni correction was made this association was not significant any more (b.p = 0.92). There was no statistically significant association with fetal presentation, placental location or AFI (Table 2).

**Table 2:** **The association between fetal factors and decreased perception of fetal movement**

Factors	Mothers with		OR	95% CI
	Perception of DFM (n = 59) N (%)	Perception of NFM (n = 670) N (%)		
Female fetuses	37 (62.7)	329 (49.1)*	1.76	1.01 – 3.05
Placental site				
● Anterior placenta	33 (55.9)	332 (49.5)	1.04	0.42 – 2.59
● Posterior placenta	19 (32.2)	261 (38.9)	0.76	0.29 – 1.99
● Other sites^◄^	6 (10.16)	63 (9.4)	-	-
Fetal presentation				
● Breech	10 (16.9)	123 (18.3)	0.9	0.44 – 1.84
● Transverse	3 (5)	17 (2.5)	1.96	0.55 – 6.96
● Cephalic^◄^	46 (77.9)	513 (76.5)	-	-
Amniotic fluid index				
● Polyhydramnios	2 (3.3)	18 (2.6)	1.27	0.27 – 5.61
● Oligohydramnios	1 (1.69)	6 (0.89)	1.9	0.22 – 16.11
● Normal^◄^	55 (93.2)	629 (93.8)	-	-

*: p < 0.05 for the comparison between two groups with and without the specified characteristic, ^◄^: reference category, OR: odds ratio, 95% CI: 95% confidence interval, DFM: Decreased fetal movements, NFM: Normal fetal movements.

### Dependency of the results

Using a multivariate logistic regression, the maternal perception of DFM remained significantly associated with maternal employment (p = 0.004), the maternal state of not having daily exercise (p = 0.009) and maternal supine position (p = 0.001). The maternal perception of DFM showed a borderline association with older maternal age (p = 0.07) and delayed perception of fetal movements (p = 0.06) (Table 3).

**Table 3:** **Multivariate logistic regression analysis of maternal and fetal factors affecting maternal perception of decreased fetal movements**

Factor	B value	Unadjusted OR (95% CI)	Adjusted OR (95% CI)	Adjusted p value
Maternal age > 35 years	0.64	2.41	1.89	0.07
		(1.28 – 4.52)	(0.94 – 3.86)	
Delayed first perception of FM	0.72	2.1	2.05	0.06
		(1.18 – 3.74)	(0.96 – 2.76)	
Maternal employment	0.98	2.67	2.66	0.004
		(1.46 – 4.9)	(1.35 – 5.23)	
Daily exercise	- 1.93	0.15	0.14	0.009
		(0.03 – 0.64)	(0.03 – 0.62)	
Maternal supine position	1.35	3.81	3.85	0.001
		(1.74 – 8.3)	(1.71 – 8.83)	
Female fetuses	0.41	1.76	1.5	0.11
		(1.01 – 3.05)	(0.79 – 2.7)	

B value: Values for the logistic regression equation for predicting the dependent variable from the independent variable, OR: odds ratio, 95% CI: 95% confidence interval, FM: fetal movements.

## Discussion

Our study showed that 8.1% of healthy normotensive singleton pregnant women with desirable pregnancy outcomes reported perception of decreased fetal movements. Almost the same rates have been reported in other studies; in an American study of 38,728 pregnancies, 6.6% were examined in the hospitals for concerns of DFM [2], In Norway maternal concern for DFM is a frequent cause for unscheduled antenatal consultations, occurring in approximately 10% of third-trimester pregnancies [4]. The overall rate of perception of DFM is reported at the range of 4% - 15% in most studies [1, 2]. In our study, some maternal and fetal factors were associated with this perception of DFM, but the three factors that remained independently associated with a perception DFM were maternal employment, the state of not having daily exercise and maternal supine position when counting the fetal movements.

A perception of DFM was reported more often by older mothers and by those who felt the first fetal movements later than the other participants. In our study, this perception was not independent of other maternal factors. In their study of maternal awareness of fetal movements, Saastad et al. indicated that maternal age ≥ 34 years was associated with a low awareness of fetal activity, but this did not affect the risk of being concerned or being examined [12].

In this study, although the maternal employment rate was low (14.4%), it was an independently associated factor with the maternal perception of DFM. Especially the women who worked more than 8 hours per day felt less fetal movements. Working mothers seem to be at an increased risk of experiencing stress and anxiety which are associated with DFM [3, 12–15]. In their study of stress in working women, Haque & Haleem indicated that working women had higher stress levels and lower serotonin levels [16], and because serotonin is linked to sustained attention [17], working mothers with low levels of serotonin could be less alert to fetal movements. It was demonstrated by Olesen & Svare in their review of fetal movements that busy mothers may not perceive as many fetal movements because they are not concentrating on fetal activity, and they often report an inaccurate reduction of fetal movements [14]. An American study documented that working pregnant women have higher catecholamine levels during the work period, and this is known to decrease uterine blood flow [18]. Reduced motor activity is the fetal response to this decreased blood flow [19]. There is an important point that requires attention regarding maternal employment in this study; in the developing countries women employment rate is lower than the developed countries; most women in the developing countries are housewives and are expected to keep their traditional roles and responsibilities at home. Therefore the working women in the developed countries are under higher pressure, due to having too many responsibilities at both work and home and may experience higher stress and anxiety than working women in the developed countries.

In the current, the study mothers who had daily exercise felt more fetal movements than the non-exercising mothers. Other studies showed a decrease in fetal breathing and body movements in response to heavy maternal exercise [9]. Exercise seems to have different effects on the maternal perception of fetal movements depending on its regularity, duration and severity. Regular mild to moderate exercise seems to improve the maternal perception of fetal movements through different mechanisms affecting both the mothers and the fetuses. Exercise results in increased serum cortisol, hippocampal dopamine and serotonin levels, and these changes lead to increased mood and mental alertness [17, 20], which can improve the maternal perception of fetal movements. A Canadian study showed that pregnant women who exercise during pregnancy experience significant decreases in depression, anger, tension, fatigue and anxiety [21], the factors which have been shown to be associated with decreased perception of fetal movements [12, 14, 15].

From the fetal perspectives, elevated maternal cortisol has been linked to increased fetal movements. A study by DiPietro et al. showed that maternal cortisol is significantly associated with fetal motor amplitude and the total time spent moving [22]. This could be due to the effect of cortisol on the developing brain; glucocorticoid exposure during the fetal period has organizational effects on the developing brain, including the modification of synaptogenesis, neurotransmitter function, and glucocorticoid receptor expression with long-term implications for structure and function [22]. In the study by Ellman et al., higher maternal cortisol levels were associated with advanced neonatal neuromuscular maturation [23]. Maternal exercise improves fetal growth and placental vascularization and increases serum placental growth factor [24, 25], which might affect fetal movements [8].

We were unable to find studies examining the effect of mother’s different positions on her perception of fetal movements. This study showed that when mothers are in the supine position, they feel less fetal movements. When a pregnant woman is in the supine position, the inferior vena cava is compressed by the gravid uterus, resulting in a decrease in cardiac output, stroke volume and ejection fraction. These changes lead to an increased heart rate [26], more suppressed vagal activity and enhanced sympathetic activity [27]. Approximately 10 – 12% of the catecholamines are transferred to the fetal circulation, which may contribute to fetal vasoconstriction and the development of fetal hypoxemia and hypoxia [28]. The fetal responses to maternal sympathetic activation and hypoxemia include increased a variability of the heart rate concomitant with the suppression of motor activity [19].

We did not find any associations between the perception of DFM and the AFI. This is consistent with a study by Almli et al., which did not find a significant association between the amniotic fluid volume and leg movements per minute [8]. Some studies have reported that decreased amniotic fluid volume is associated with DFM [5]. A review of DFM indicated that oligohydramnios and polyhydramnios have been shown to be associated with DFM [14]. Several factors can account for these differences, including interobserver and intraobserver variations [29], gestational age at the examinations, sample size and the percentage of abnormal AFI in these studies.

Studies with conflicting results have assessed the associations of the placental site and the perception of DFM. In the present study, no significant association was found between the perception of DFM with the placental location, and similar results were reported by several smaller studies (a total of 94 women) [10], a British study of 182 patients reported an association of DFM with an anterior placenta [6]. In another study of 284 women, an anterior placenta was associated with a reduced perception of fetal movements only in some gestational ages and not throughout the entire pregnancy [30]. These conflicting results are mainly due to the small sample sizes, different gestational ages and lack of consistent definitions [10].

This study is among the few studies to search for an association between DFM and fetal presentation. In our study, the mothers of fetuses with transverse presentation had a higher percentage for the perception of DFM, but this difference was not statistically significant. A Canadian study of 28 fetuses indicated that there are no differences in the spontaneous fetal heart rate, body or breathing movements in fetuses in the breech position compared with the cephalic position, but fetuses in the breech position showed atypical movement responses to vibroacoustic and airborne sound stimuli [7]. Sherer et al. in their study of 465 pregnant women, reported that the fetal presentation was not significantly different between the patients based on the score of fetal movements [5].

One of the main limitations of the study was the compliance of the participants; due to the limited compliance of the participants we could not ask them to count the fetal movements till the end of pregnancy to see if any changes happen in maternal perception of fetal movements over time. Another limitation of the study was the small number of participants who had some of the studied characteristics, for instance we could not have an accurate assessment of the association of maternal perception of DFM with oligohydramnios.

## Conclusions

This study shows that 8.1% of healthy normotensive pregnant women who give birth to healthy term newborns report perception of decreased fetal movement when asked to count their fetal movement during the third gestational trimester which is independently associated with maternal employment, supine position when counting fetal movement and not having daily exercise. These factors should be taken into consideration when using this screening tool for assessing fetal well-being and also should be considered for the interpretation and management of patients presenting with concerns related to a decreased perception of fetal movement.
